# Parotid Gland Carcinoma Masquerading as an Aural Polyp

**Published:** 2019-09

**Authors:** Sethu-Thakachy Subha, Saraiza Abu-Bakar, Narayanan Prepageran

**Affiliations:** 1 *Department of Otorhinolaryngology, Head & Neck Surgery,* *University Putra Malaysia (UPM), Selangor, Malaysia.*; 2 *Department of Otorhinolaryngology, Head & Neck Surgery, Hospital Serdang, Selangor, Malaysia.*; 3 *Department of Otorhinolaryngology, Head & Neck Surgery, Faculty of Medicine, University Malaya, Malaysia.*

**Keywords:** Aural polyp, Parotid tumor, Primary squamous cell carcinoma

## Abstract

**Introduction:**

Parotid gland squamous cell carcinoma is an uncommon aggressive neoplasm with poor prognosis. Aural polyps are usually the presenting features of chronic suppurative otitis media, tuberculous otitis media, and adenoma or carcinoma. The malignant aural polyp is very rare. Parotid gland carcinoma masquerading as an aural polyp has rarely been described in the literature.

**Case Report:**

We report a case study of parotid squamous cell carcinoma in a 29-year-old male masquerading as an ear polyp.

**Conclusion:**

Parotid gland primary squamous cell carcinoma is a rapidly advancing neoplasm which carries poor prognosis despite multimodality treatment. Diligent clinical and histopathological evaluation is imperative to discriminate this rare aggressive disease from the metastatic and other primary cancers of the parotid. A high index of suspicion is crucial in refractory aural polyps to arrive at early diagnosis.

## Introduction

Parotid gland primary squamous cell carcinoma is an extremely unusual malignancy comprising of less than 1% of all parotid tumors (1-3). Generally, the diagnosis of parotid gland carcinoma is established after excluding the high-grade mucoepidermoid carcinoma and invasion or metastases from an extra-parotid source (1,2,4,5). We report an unusual case of parotid gland primary squamous cell carcinoma mimicking as an aural polyp. The clinical progress, recurrence, and metastasis of this entity are difficult to prognosticate due to its low incidence and heterogeneous histological manifestations (6). Early detection of this rare rapidly advancing lesion is of utmost importance in providing adequate treatment.

## Case Report

**Fig 1:**
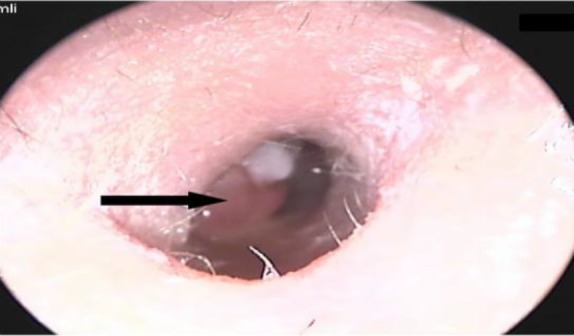
Polypoid lesion in the external auditory canal

A 29-year-old male attended a general practitioner’s clinic with right ear pain, discharge and reduced hearing for many months. The patient felt a lesion and attempted to remove it himself. Subsequently, he developed bleeding from his right ear. He went to see a general practitioner who referred him to the Ear Nose and Throat outpatients’ clinic. The general examination showed a clinically stable patient with no neurological deficits. Examination of the right ear revealed purulent ear discharge with a polypoid lesion in the external auditory canal partly obscuring the tympanic membrane (Fig.1). A clinical diagnosis of ear polyp secondary to chronic otitis media was made in this study. Oral antibiotics and topical steroid drops were given and the patient was advised to refrain from self-cleaning. During his follow up visit, he was diagnosed with right postauricular abscess and admitted for intravenous antibiotics. 

**Fig 2:**
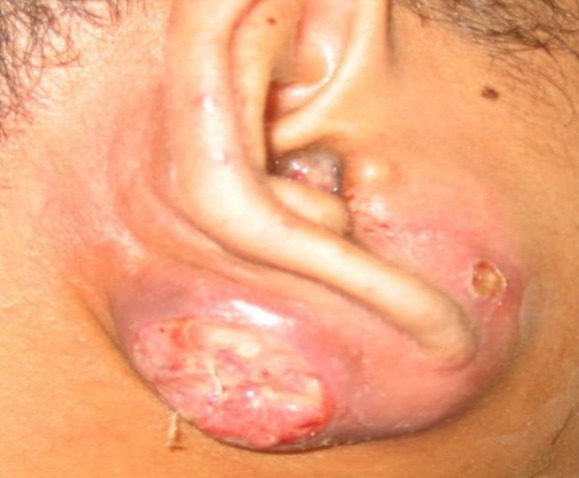
Fungating mass at the postauricular region

A temporal computed tomographic (CT) scan demonstrated the presence of a mass in the external ear canal extends into parotid space and right parapharyngeal space with intact middle ear structures. Magnetic resonance imaging (MRI) established similar findings with no intracranial extension. Biopsy of the polyp revealed to be squamous cell carcinoma. He refused for any treatment initially and subsequently he presented with right-sided facial palsy and fungating mass at the postauricular region extending into the infra-auricular region (Fig.2). 

There were no palpable cervical lymph nodes. The patient underwent right radical parotidectomy and sacrifice of the facial nerve. The tumor was found intraoperatively to be arising mainly from the deep lobe of the parotid gland involving the anteroinferior part of ear canal extending medially into infratemporal fossa and para pharyngeal space, sparing the posterior canal wall and middle ear. The facial nerve was surrounded by a tumor. Microscopically, the malignant squamous epithelial cells showed pleomorphic vesicular nuclei with prominent nucleoli and bizarre mitotic figures. 

The microscopic section also exhibited prominent individual cell keratinization and keratin pearls. There was no evidence of lymphovascular permeation and intraparotid lymph nodes infiltrations. Therefore, histopathology was confirmed as well differentiated squamous cell carcinoma. The patient was referred for further oncological treatment. Unfortunately, he defaulted the follow-up.

## Discussion

Parotid gland squamous cell carcinoma is a rare primary malignancy with a high mortality rate (1-4,7). Primary squamous cell carcinoma is regarded as a diagnosis of exclusion after a history of metastasis from a distant primary carcinoma or high-grade mucoepidermoid carcinoma (1,3). A retrospective study by Batsakis et al. have demonstrated that the incidence of parotid gland primary squamous cell carcinoma is lower than that of metastatic carcinoma (2).

The ionizing radiation is the highly speculated potential risk factor for parotid squamous cell carcinoma (1,5,6). However, there was no record of irradiation to the salivary gland in our patient under study. Regarding the incidence of parotid primary squamous cell carcinoma, males are more commonly affected than females. Although the age group of the patients affected varying from 48 to 86 years, most of them were 65 years or over (6-8).

The usual manifestation of primary squamous cell carcinoma is the rapidly enlarging mass around the angle of the mandible with facial nerve weakness and cervical lymphadenopathy (1-4,7). The mass may be painful, fixed to the skin or the deeper structures. Lee et al. reported that these lesions were rapidly advancing, diffusely invading the parotid gland and its adjacent structures (4). 

The aetiology of aural polyp can be varied from inflammation to malignancy. Therefore, it is mandatory to obtain origin, extent, and histological diagnosis in all polyps to provide definitive treatment (9). Imaging techniques, such as CT scan and MRI can add to diagnostic precision in mapping the extension and delineate the depth of ear polyps (10,11). 

Salivary gland tumors always pose problems in diagnosis due to their infrequency and variation in histology (6). The histogenesis of this lesion is controversial and it probably originates from metaplastic duct epithelium (2,4). Most of the described parotid primary squamous cell carcinoma have been well or moderately differentiated carcinomas (2,7). The histopathological finding of our patient was also in accordance with the literature. The classic histological features of parotid primary squamous cell carcinoma are intracellular keratinization and keratin pearl formation. These features may assist in distinguishing primary squamous cell carcinoma from high grade mucoepidermoid and metastatic carcinomas (2).

Parotid squamous cell carcinoma does not have any typical growth progress, clinical features, and metastasis (3,5). Batsakis et al. in their retrospective study reported that local recurrence and metastatic lymphadenopathy were the general outcomes for the patients with the parotid gland primary squamous cell carcinoma. Distant metastasis is very rare (8). Lee et al. in their study observed that increased risk of loco-regional recurrence in late stages, large lesion, facial nerve paralysis, and metastatic lymphadenopathy during the initial clinical presentation (4). In their study, they also found that over 60 years of age was the statistically significant unfavorable prognostic factor for disease-free survival. The overall survival of primary squamous cell carcinoma was 50% (3,7,8). 

According to the obtained data, the prognosis of parotid primary squamous cell carcinoma is poor, compared to that in patients suffering from squamous cell carcinoma in other sites (2- 4). Due to the paucity of this tumor, there is a lack of adequate published data regarding the treatment options of parotid squamous cell carcinoma (2-4). Various studies indicated the radical resection and postoperative radiotherapy as the combined treatment in parotid squamous cell carcinoma (1-4). Moreover, the extent of surgical resection is defined by tumor involvement. Local recurrence and regional metastasis are the end results for these patients despite the fulfillment of various treatment strategies (2).

## Conclusion

Parotid gland primary squamous cell carcinoma is an aggressive neoplasm with poor prognosis. Though parotid gland primary squamous cell carcinoma is a rare entity, it can masquerade as an aural polyp. Careful clinical and histopathological examination is of utmost importance in early diagnosis and appropriate treatment to be instituted.
